# Allograft Inflammatory Factor-1 Governs Hematopoietic Stem Cell Differentiation Into cDC1 and Monocyte-Derived Dendritic Cells Through IRF8 and RelB *in vitro*

**DOI:** 10.3389/fimmu.2019.00173

**Published:** 2019-02-08

**Authors:** Diana M. Elizondo, Nailah Z. D. Brandy, Ricardo L. L. da Silva, Naomi L. Haddock, Apollo D. Kacsinta, Tatiana R. de Moura, Michael W. Lipscomb

**Affiliations:** ^1^Department of Biology, Howard University, Washington, DC, United States; ^2^Laboratório de Imunologia e Biologia Molecular-Hospital Universitário, Universidade Federal de Sergipe, Aracaju, Brazil; ^3^Immunology Program, Stanford University, Stanford, CA, United States; ^4^Department of Cellular and Molecular Medicine, UCSD School of Medicine, La Jolla, CA, United States

**Keywords:** dendritic cells, allograft inflammatory factor 1, hematopoietic stem cells, cDC1 dendritic cells, monocyte-derived DC, RelB/NFκB, IRF8 transcriptional coactivator, protein kinase C (PKC)

## Abstract

The multistep differentiation process from hematopoietic stem cells through common myeloid progenitors into committed dendritic cell (DC) subsets remains to be fully addressed. These studies now show that Allograft Inflammatory Factor-1 (AIF1) is required for differentiation of classical DC type 1 (cDC1) subsets and monocyte-derived DC (Mo-DC). Phenotypic studies found that AIF1 expression increased in committed subsets differentiating from common myeloid progenitors (CMP). However, silencing AIF1 expression in hematopoietic stem progenitors restrained the capacity to differentiate into Mo-DC and cDC1 cell subsets under GM-CSF or Flt3-L stimuli conditions, respectively. This was further marked by restrained expression of IRF8, which is critical for development of Mo-DC and cDC1 subsets. As a result, absence of AIF1 restrained the cells at the Lin^−^CD117^+^FcγR^−^CD34^+^ CMP stage. Further biochemical studies revealed that abrogating AIF1 resulted in inhibition of the NFκB family member RelB expression and p38 MAPK phosphorylation during differentiation of Mo-DC. Lastly, protein binding studies identified that AIF1 interacts with protein kinase C (PKC) to influence downstream signaling pathways. Taken together, this is the first report showing a novel role of AIF1 as a calcium-responsive scaffold protein that supports IRF8 expression and interacts with PKC to drive NFκB-related RelB for successfully differentiating hematopoietic progenitor cells into cDC and Mo-DC subsets under Flt3-L and GM-CSF stimuli, respectively.

## Introduction

Dendritic cells (DC) survey the microenvironment by capturing, processing, and presenting antigens to promote adaptive immunity (1). Hematopoietic stem cells (HSC) can be directed into either common myeloid progenitors (CMP) or common lymphoid progenitors (CLP) to give rise to diverse DC subsets (2). Furthermore, monocytes, macrophages and DC are all derived from the common monocyte-macrophage DC progenitor (MDP). Importantly, this subset can direct generation of the common DC progenitor (CDP), which can uniquely give rise to diverse DC subsets, but not to macrophages, monocytes or other granulocyte populations (3, 4). Alternatively, MDP can also direct fates toward common monocyte precursors (cMoP), which can differentially give rise to monocytes that, under diverse inflammatory stimuli, generate monocyte-derived DC (Mo-DC), or macrophages. Although DC developmental processes have been extensively studied (5), the underlying mechanisms involving other fate-determining genes remain to be delineated.

The differentiation pathway of DC subsets from hematopoietic progenitors can be driven by granulocyte macrophage-colony stimulatory factor (GM-CSF; Csf-2) (6) or Fms-Related Tyrosine Kinase 3-Ligand (Flt3-L) cytokines (7–9). *In vivo* and *in vitro* Flt3-L-derived subsets are further divided into classical (*or conventional*) dendritic cells (cDC), which can be distinguished by the specific expression of the Zbtb46 transcription factor (10), or plasmacytoid DC (pDC). CD8α^+^/CD24^+^/CD103^+^ cDC1 subsets are directed by IRF8 (11, 12), with concerted activities of the BATF3 transcription factor being required (13). Whereas, the CD11b^+^/SIRPα^+^ cDC2 are not dependent on IRF8, but still require IRF4 (14). Flt3-L stimulation *in vitro* can drive cDCs with profile and functions that resemble that of splenic subsets (15). GM-CSF has been shown to be crucial for promoting survival, proliferation, and homeostasis of non-lymphoid DC (6, 16). *In vitro*, it has been shown to expand a heterogeneous pool of Mo-DC and macrophages that expresses CD11c and MHC class II (6, 17), with Mo-DC being more proficient in cross-presenting soluble antigens than the generated macrophages (18). However, the *in vivo* cross-presentation role remains unclear with respect to antigen presentation and direction of cognate T cell responses.

The NFκB family signaling activities can directly regulate immune cell differentiation and responses. Activation of the NFκB pathway can be driven by the recruitment of protein kinase C (PKC) (19). Furthermore, the NFκB family member RelB directs DC development *in vivo* (20–22), albeit different from its observed role for *in vitro* generation (23). In addition to development, RelB is important in controlling MHC class II expression and maturation of DC (24). Importantly, RelB is directly suppressed by the activated states of IκB, which is an inhibitor of NF-κB proteins (24). From the MAPK pathway, the three most characterized members are ERK, JNK/SAPK, and p38 Kinase. Interestingly, p38 MAPK is important for regulating NFκB recruitment to nuclear targets (25).

Allograft Inflammatory Factor-1 (AIF1), also known as ionized-calcium binding adapter molecule 1 (Iba1), is a 17 kD interferon gamma-inducible calcium binding EF-hand protein (26). The gene has shown diverse roles in the nervous and immune systems (27, 28). In particular, AIF1 expression in activated macrophages, microglial cells and DC plays major immunomodulatory roles during inflammatory responses (26, 29, 30). Although the importance of AIF1 in antigen presentation by DC has been reported (29), no study has delineated its role in differentiation.

This report now shows that AIF1 expression in GM-CSF- or Flt3-L-stimulated hematopoietic progenitors is required for differentiation into Mo-DC and cDC1 subsets, respectively. Under Flt3-L stimuli, loss of AIF1 resulted in restrained IRF8, BATF3, RelB, and Zbtb46 expression, but not PU.1 or Id2. Interestingly, there was a greater ratio of observed cDC2 subsets. For Mo-DC, loss of AIF1 during differentiation under GM-CSF stimuli resulted in restrained CIITA, IRF8, IRF4, and RelB. Collectively, the studies revealed that absence of AIF1 alters *in vitro* differentiation of DC away from cDC1 and Mo-DC fates.

## Materials and Methods

### Animals

All animal procedures were performed in accordance and approved by the Institutional Animal Care and Use Committee. Mice were purchased from The Jackson Laboratory (Bar Harbor, ME) and housed in pathogen-free facilities at Howard University. C57BL/6 (wild type; WT) male and female mice 8–12 weeks of age were used as a source of bone marrow and spleen.

### Generation of Monocyte-Derived Dendritic Cells (Mo-DC)

Mo-DC were generated as described by a modified protocol of Inaba et al. (17). Briefly, bone marrow cells from murine tibias and femurs were passed through a 70 μm nylon mesh to remove debris. Total isolated bone marrow cells were cultured in IMDM (Thermo Fisher; Grand Island NY) supplemented with 10% fetal bovine serum (FBS; Gibco), 2 mM L-glutamine (Gibco), 100 U/mL penicillin/streptomycin (Gibco), and 20 ng/mL recombinant mouse GM-CSF (Peprotech; Rochy Hill NJ) for 7–8 days in culture.

### Generation of Classical Dendritic Cells (cDC)

Briefly, bone marrow (BM) from murine tibias and femurs were passed through a 70 μm nylon mesh to remove debris. The isolated cells were treated with red blood lysis buffer. Total isolated bone marrow cells were then cultured in IMDM (Thermo Fisher; Grand Island NY) supplemented with 10% fetal bovine serum (FBS; Gibco), 2 mM L-glutamine (Gibco), 100 U/mL penicillin/streptomycin (Gibco) 200 ng/mL of murine Flt3-L (Peprotech) for 8–9 days in culture.

### Sorting of Lin^−^CD117^+^ Cells

Isolated bone marrow (BM) cells were incubated with a lineage (Lin) antibody cocktail conjugated to magnetic beads (Miltenyi, Auburn CA). The cocktail included: anti-mouse CD3t, CD45R (B220), CD11b, Gr-1 (Ly-6G/C), and Ter119. After staining, cells were passed over a depletion column affixed to a magnet (Miltenyi). Flow through cells are lineage negative (Lin^−^). These Lin^−^ cells were subsequently stained with CD117 antibody conjugated to magnetic microbeads (Miltenyi). After incubation, cells were passed through a collection column affixed to a magnet. The CD117 positive fraction was collected; these are the Lin^−^CD117^+^ cells.

### siRNA and Electroporation

siRNA oligonucleotide (oligo) with the following sequence was used for AIF1 knockdown: 5′-CGUUCUCUAGACGGUAGAAC-3′ (siAIF1), as previously published by Elizondo et al. (29). Scrambled siRNA control oligo used was 5′-CCUAUAGAUACCGAGUGGUTT-3′. Total BM or Lin^−^CD117^+^ sorted cells were transfected with 1.5 nanomoles of siRNA on days 0, 2, and 4 of the DC differentiation process in 4 mm electroporation cuvettes using an ECM 830 square wave electroporator (Harvard Apparatus, Holliston MA) (31). The following settings were used: 310 V, 10 ms, 1 pulse.

### CRISPR-Mediated Gene Silencing and Electroporation

The CRISPR Cas9 system was used to knockout AIF1 in Lin^−^CD117^+^ HSC or total BM. Two CRISPR DNA plasmids were created using the GeneArt CRISPR Nuclease Vector Kit (Thermo Fisher) to target AIF1. The gRNA sequences were: 5′-AGAGTAGCTGAACGTCTCCT-3′ (pAIF1-T3) and 5′-GCTGAAGAGATTAATTAGAG-3′ (pAIF1-T4). Control plasmids contained scrambled targeting sequences (pControl). Plasmids were purified using PureLink™ HiPure Plasmid Maxiprep Kit (Thermo Fisher) and cleaned with the MiraCLEAN® Endotoxin Removal Kit (Mirus, Madison WI). Twenty microgram of control or AIF1 targeting plasmids were electroporated using a square wave electroporator under the following conditions: 230 V, 4 ms, 5 pulses.

### qPCR

To evaluate gene expression, cells were harvested and resuspended in Trizol (Thermo Fisher Scientific) prior to total RNA extraction. Total RNA was reverse transcribed into single-stranded cDNA using the High Capacity cDNA Reverse Transcription Kit (Thermo Fisher Scientific). For quantitative PCR reactions, Gene Expression Master Mix, and the following probes purchased from Thermo Fisher Scientific were used: AIF1 (Mm00479862_g1), BATF3 (Mm01318274_m1), CD11c (ITGAX; Mm00498701_m1), CIITA (Mm00482914_m1), Id2 (Mm00711781_m1), IRF8 (Mm00492567_m1), IRF4 (Mm00516431_m1), MHC class IIα (H2-Aα; Mm00439216_m1), MHC class IIβ (H2-Aβ; Mm00439211_m1), PU.1 (SPIB1; Mm00488140_m1), RelB (Mm00485664_m1), and Zbtb46 (Mm00511327_m1). Samples were ran on the QuantStudio 5 real time-PCR system (Thermo Fisher). Expression levels of the target transcripts were calculated by the comparative Ct method (2–ΔΔCt formula) after normalization with the housekeeping genes GAPDH (Mm99999915_g1) and β-actin (Mm02619580_g1).

### Antibodies and Live/Dead Staining Dye

AIF1 (EPR16588) antibody used was purchased from Abcam (Cambridge MA). CD117 (2B8), Lineage cocktail (CD3/Gr-1/CD11b/CD45R/TER-119), SCA1 (D7), CD34 (SA376A4), CD11c (N418), MHC class II (M5/114.15.2), CD8α (53–6.7), CD11b (M1/70), F4/80 (BM8), CD45R (RA3-6B2), TCRβ (H57-597), Protein Kinase C (PKC; PKC0103), CD4 (RM4-5), CD3 (17A2), IgD (11-26c.2a), CD19 (6D5), CD24 (M1/69), PDCA1 (927), CD172α (P84), IL-7Rα (A7R34), CD135 (A2F10), and CD103 (2E7) purchased from BioLegend. RelB (C1E4), NFκB p100/P52 (D7A9K), phospho-IκBα (S32), IκBα (L35A5), phosho-p38 (M139), and p38 (D13E1) purchased from Cell Signaling Technology (Danvers MA). Phospho-ERK (M206) purchased from ECM biosciences (Versailles KY) and IRF4 (343), IRF8 (V3GYWCH), NFκB p105/50 (catalog #14-6732-81), GAPDH (GA1R), and β-actin (BA3R) from Thermo Fisher. Zbtb46 (U4-1374) purchased from BD Biosciences (San Jose CA). Live/Dead fixable dead cell stain (cat# L23101) purchased from Thermo Fisher Scientific.

### Western Blot Analysis

Cells were treated with NP-40 lysis buffer supplemented with a protease and phosphatase inhibitor cocktail (Amresco, Solon OH). Lysates were ran in 10–15% SDS-PAGE gels. Protein content was transferred to nitrocellulose blots by wet tank transfer. Membranes were blocked in 5% non-fat dry milk or 2% BSA prior to primary antibody staining. For phospho-specific antibodies, probing for total respective protein content was used for quantitative profiling. After primary antibody staining, secondary fluorochrome-conjugated antibodies were used for detecting protein bands on the Licor Odyssey imaging system (Licor, Lincoln NB). Datasets were analyzed using Image Studio 5.2 software (Licor).

### Co-immunopreciptation

Lysates were prepared using NP-40 protein lysis buffer supplemented with a protease and phosphatase inhibitor cocktail. One hundred microgram of protein lysates were incubated with 5 μg AIF1 or IgG rabbit control antibody. Next, rec-Protein G-Sepharose® 4B conjugate beads (Thermo Fisher) were re-suspended with the protein lysates coupled with antibody. After incubation, samples were extensively washed and centrifuged at 6,000 × g for 30 s. The pelleted antibody-protein bead complex was then resuspended in 10% SDS diluted in NP-40 lysis buffer. Samples were then ran in 10% SDS-PAGE gels and transferred to a nitrocellulose membrane using wet tank transfer. Membranes were blocked with 2% BSA prior to probing with PKC antibody. Membranes were then washed prior to probing with secondary fluorochrome-conjugated antibodies and detected using the Licor Odyssey imaging system.

### Cryosectioning and Fluorescence Microscopy

Mice spleens were harvested and immediately cryosectioned at 10 or 20 μm thickness prior to fixation with 3% paraformaldehyde. Samples were transferred onto glass slides prior to permeabilization using 0.3% Triton-X solution and blocking with 0.2% BSA. Sections were stained with AIF1 or IgG isotype control antibodies, followed by washing and secondary staining. Additionally, sections were co-stained with CD11c, CD4, F4/80, or CD45R/B220. DAPI (Thermo Fisher) was used as a nuclear staining dye. After incubation, slides were then washed prior to mounting with cover slips and imaging using the FSX100 fluorescence microscope (Olympus, Waltham MA). Acquired images were then analyzed using ImageJ (Rasband, W.S., ImageJ, U. S. National Institutes of Health).

### Flow Cytometry

Cell surface staining was performed with PBS supplemented with 1 mM EDTA, 0.1% NaN_3_ and 2.5% bovine serum (FACS buffer). Cells were washed with FACS buffer prior to extracellular staining with fluorochrome-tagged antibodies. Dilutions were antibody specific, but roughly 10 μl of a 10 μg/mL working concentration was utilized per 2 × 10^5^ cells. Respective isotype controls were used in all assays. Cells were then fixed with 3% PFA in PBS. For intracellular antibody labeling, fixed cells were permeabilized with 0.2% saponin in PBS with 1 mM EDTA. Next, primary antibodies or isotype controls were added at ~10 μg/mL concentrations followed by washing and subsequent staining with secondary fluorochrome-labeled antibodies. Cells were acquired on a BD FACSVerse flow cytometric analyzer (BD Biosciences). Datasets were analyzed using Flow Jo v10 (Flow Jo LLC; Ashland, OR).

### Statistical Analysis

GraphPad Prism v8.0 (GraphPad Software, La Jolla CA) was used to determine statistical significance. Student unpaired two-tailed *t-*test was used to evaluate the significance between two groups. Error bars for all figures indicate standard errors; ^*^< 0.05, ^**^< 0.01 and NS = not significant.

## Results

### AIF1 Is Restricted to Subsets of Hematopoietic Progenitors and Myeloid-Derived Lineage Committed Cells

**Figure 1:**
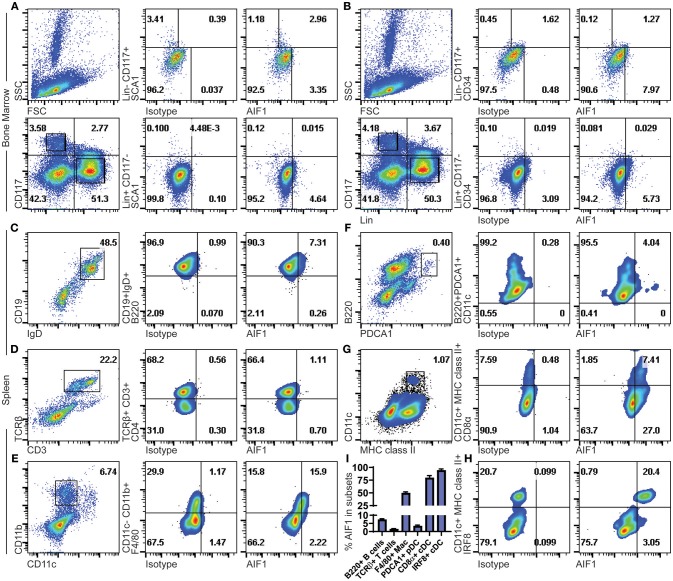
AIF1 is expressed in Lin^−^CD117^+^SCA1^+^ bone marrow hematopoietic stem cells and in spleen myeloid-derived subsets. Bone marrow cells were assessed for hematopoietic subsets. Dot plots show SSC vs. FSC and Lineage (Lin) vs. CD117 (c-Kit) populations. Expression of AIF1 was assessed in Lin^−^CD117^+^ (top row) and Lin^+^CD117^−^ (bottom row) cells co-expressed with either **(A)** SCA1 or **(B)** CD34. Splenocytes were evaluated for AIF1 expression in subsets of B cells, T cells, macrophages, plasmacytoid DC, and conventional DC; cell populations were, respectively, identified by: **(C)** CD45R (B220)^+^ gated from IgD^+^CD19^+^ cells; **(D)** CD4^+^ subsets gated from CD3^+^TCR^+^ cells; **(E)** F4/80^+^ subsets gated from CD11c^−^CD11b^+^ cells; **(F)** CD11c^+^ subsets gated on B220^+^PDCA1^+^; and **(G)** CD8α^+^ gated from MHC class II^+^CD11c^+^ conventional dendritic cells. **(H)** IRF8^+^ cells gated from CD11c^+^MHC class II^+^ subsets. **(I)** AIF1 expression percentage in B220^+^ B cells gated from CD19^+^IgD^+^ subsets, TCRβ^+^ T cells, F4/80^+^ macrophages gated from CD11c^−^CD11b^+^ subsets, PDCA1^+^ pDC, CD8a^+^ cDC gated from CD11c^+^MHC class II^+^ subsets, and IRF8^+^ cDC gated from CD11c^+^MHC class II^+^ subsets. All gates were established using respective isotype controls. Isotype used for determining AIF1 expression is shown in all groups. Data is representative of three independent experiments.

AIF1 expression was assessed in murine bone marrow hematopoietic progenitors. Gated populations of Lin^−^CD117^+^ and Lin^+^CD117^−^ bone marrow cell populations were assessed for AIF1 in conjunction with SCA1 or CD34 co-expression. In all studies, isotype controls were used to establish gating strategies and compensate for differential levels of Fc receptors pinocytosis on the heterogenic pool of bone marrow cells. Results showed AIF1 expression at 2.96% ± 0.08 in the Lin^−^CD117^+^SCA1^+^ hematopoietic subsets (Figure 1A), which represents 73.4% ± 4.3 of the population positive for AIF1. Lower levels of AIF1 were found in the Lin^+^CD117^−^SCA1^−^ bone marrow subsets at 4.64% ± 0.4. Studies found no co-expression of AIF1 with CD34 in either Lin^−^CD117^+^ or Lin^+^CD117^−^ subsets (Figure 1B).

Next, AIF1 was evaluated in murine splenocytes. For lymphocytes, B cells were assessed by gating on CD19^+^IgD^+^ subsets to evaluate CD45R(B220) co-expression with AIF1 (Figure 1C). Results revealed a small proportion of B cells that were reproducibly found to express AIF1. For T cells, splenocytes were gated on CD3^+^TCRβ^+^ to evaluate CD4^+^ T cells (Figure 1D). No expression was found in TCRβ^+^ T cells. Myeloid subsets, macrophages, plasmacytoid DC (pDC), and classical DC were assessed for AIF1 expression. Results found that the CD11b^+^CD11c^−^F4/80^+^ macrophage subsets at 15.9% ± 0.4, representing about 50% of the population (Figure 1E). Analysis of CD11c^+^B220^+^PDCA1^+^ pDC showed little-to-no expression with 4.04% ± 0.5 cells expressing AIF1 (Figure 1F), nor in the alternative gating of B220^+^CD11b^−^Gr1^+^CD11c^+/lo^ pDC (Supplemental Figure 1A). However, in assessing the classical DC, results revealed high levels of AIF1 expression in the CD11c^+^MHC class II^+^ population (Figure 1G). Interestingly, the CD11c^+^MHC class II^+^CD8α^+^ cDC1 subsets dominantly expressed AIF1 at 7.4% ± 0.3 compared to 1.8% ± 0.2 not expressing the protein (Figure 1G). This represented 80% of the cDC1 population expressing AIF1, compared to only about 30% of AIF1 expressed in the CD8α^−^ subsets. To further assess cDC1 profile, studies evaluated co-expression of IRF8 with AIF1 in the CD11c^+^MHC class II^+^ subsets. Results revealed that all CD11c^+^MHC class II^+^IRF8^+^ cDC expressed AIF1 (Figure 1H). Further analysis revealed 21.6% ± 0.5 AIF1 expression in IRF4^+^ cells and 21.2% ± 0.6 in Zbtb46^+^ cells (Supplemental Figures 1B,C). Collectively, AIF1 is predominately present in myeloid splenic subsets, with the most expression found in CD11c^+^MHC class II^+^ cDC1 population, and a lesser fraction in the CD11b^+^CD11c^−^F4/80^+^ macrophages (Figure 1I).

**Figure 2:**
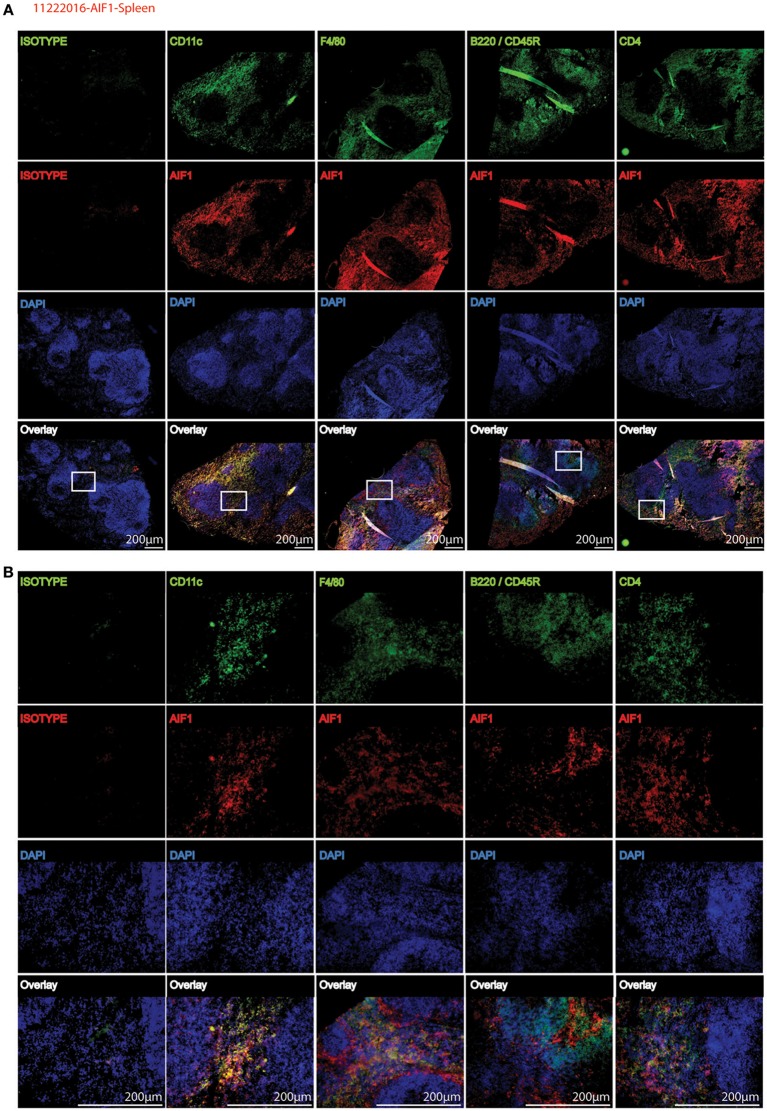
AIF1 is dominantly expressed in splenic myeloid subsets. Fluorescence microscopy was performed on 20 μm spleen sections evaluating AIF1 expression (red) co-stained for CD11c, F4/80, CD45R (B220), or CD4 (green) with respective isotype controls. DAPI was used to stain for nuclei (blue). Stained sections were imaged at **(A)** 4X and **(B)** 20X magnifications. Sections in the white box area of 4X images (top) represents respective magnified 20X images (below). Images were captured on an Olympus FSX100 fluorescence microscope. Data is representative of three independent experiments.

To corroborate flow cytometric analyses, spleen sections were stained to determine co-localization of AIF1 with common CD11c, F4/80, B220, and CD4 myeloid and lymphoid cell markers. Images are presented at 4X magnification, with a cropped section further magnified to 20X (Figures 2A,B). Results revealed that AIF1 was predominately co-expressed in the myeloid-associated CD11c^+^ and F4/80^+^ subsets. Little-to-no co-localization was observed in the B220^+^ or CD4^+^ cell subsets, which are markers largely associated with lymphocytes.

### Increase in AIF1 Expression Over Time Is Directly Associated With DC Differentiation by Either GM-CSF or Flt3-L Stimulation

**Figure 3:**
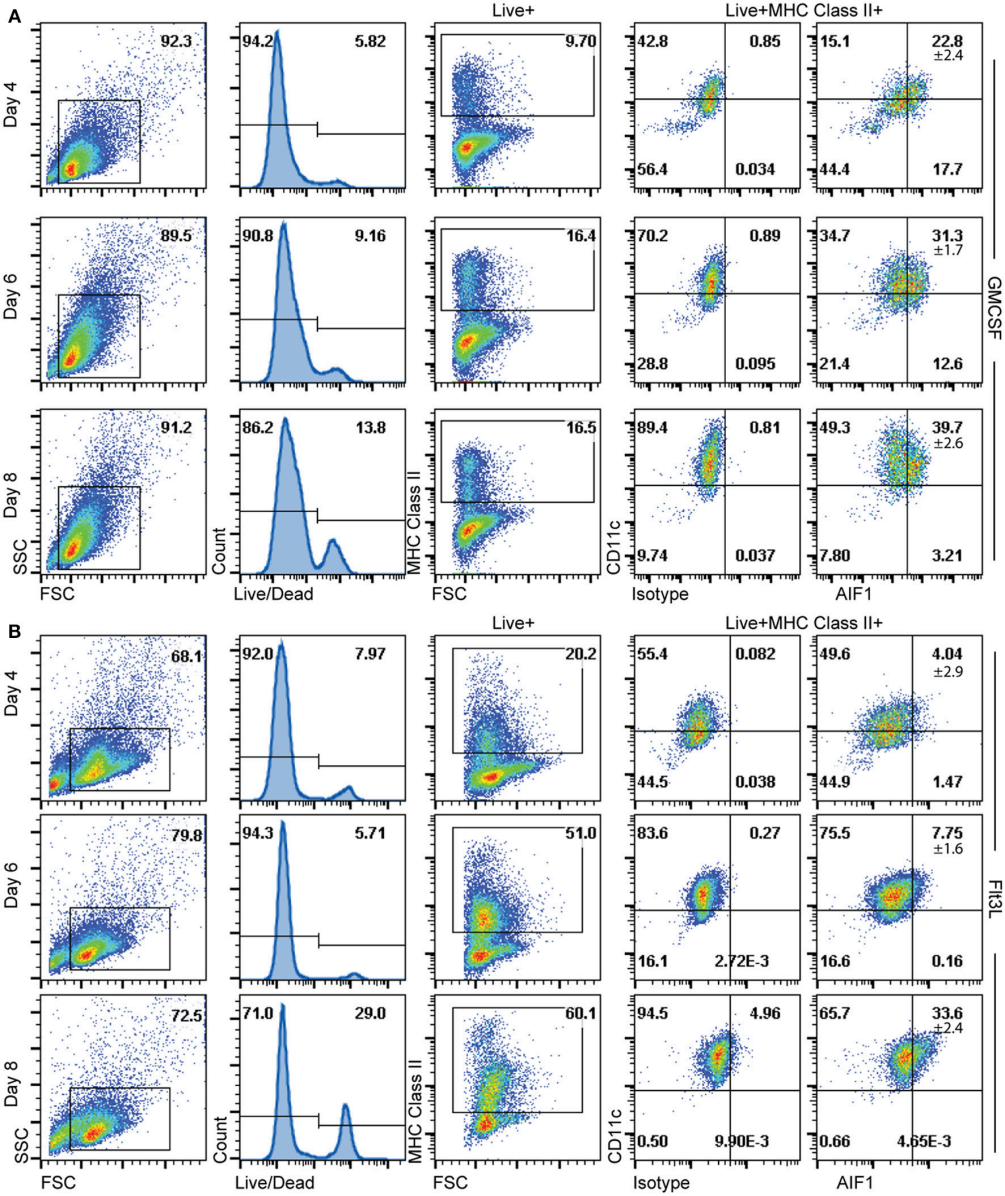
AIF1 expression during GM-CSF or Flt3-L DC differentiation from bone marrow-derived cells. Differentiating DC were harvested on days 4, 6, and 8 of **(A)** GM-CSF or **(B)** Flt3-L stimulated bone marrow cells. Live cells were gated out by using live/dead fixable dyes; dead cells are preferentially labeled with the staining dye to discriminate from the live population. MHC class II^+^ subsets were then gated from the Live^+^ population. AIF1 expression was then assessed in CD11c^+^ subsets gated from MHC class II^+^ groups. All gates were established using isotype controls. Data is representative of five independent experiments.

Having determined that AIF1 is largely present in CD11c^+^MHC class II^+^ subsets, the studies next investigated the time point(s) to which AIF1 appears during DC differentiation under GM-CSF or Flt3-L stimuli from murine bone marrow *in vitro*. Cells were then harvested on days 4, 6, and 8 and stained for flow cytometric analyses. Staining with live/dead exclusion dye was used to preferentially stain dead cells and identify the live population. These Live^+^ cells were then gated to assess AIF1 expression in CD11c^+^MHC class II^+^ subsets. Results revealed that AIF1 levels progressively increased in the DC throughout the 8-day differentiation process under GM-CSF (Figure 3A) or Flt3-L (Figure 3B). For GM-CSF stimuli, there was a day-by-day increase in AIF1 within the CD11c^+^MHC class II^+^ subsets, with day 4 at 22.8% ± 2.4, day 6 at 31.3% ± 1.7 and day 8 at 39.7% ± 2.6. Similar to GM-CSF-treated cultures, Flt3-L stimulated cultures showed that AIF1 increased throughout the differentiation, with day 4 at 4.04% ± 2.9, day 6 7.75% ± 1.6, and day 8 33.6% ± 2.4 within the CD11c^+^MHC class II^+^.

### Differential Expression of AIF1 in DC Subsets Derived From Spleen or Expanded *in vitro*

For further analyses, myeloid subsets derived from the spleen or *in vitro* expanded from bone marrow precursors under either GM-CSF- or Flt3-L-stimuli were co-stained for MHC class II, CD11c, CD8α, CD11b, and CD103. Results revealed that the majority of the CD11c^+^MHC class II^+^CD8α^+^ cDC1 subsets express AIF1, with considerably lower levels found in CD11b^+^ cDC2 (Supplemental Figures 2A–C). As expected, no CD103^+^ myeloid DC were detected in the spleen. There was relatively low levels of AIF1 present in MHC class II^+^CD11c^−^CD11b^+^ subsets, whereas the highest expression was in the CD11c^+^CD11b^−^ populations (Supplemental Figures 2D–F).

### Silencing AIF1 in Bone Marrow Cells Inhibits *in vitro* Differentiation of CD11c^+^ DC Subsets in the Presence of GM-CSF

**Figure 4:**
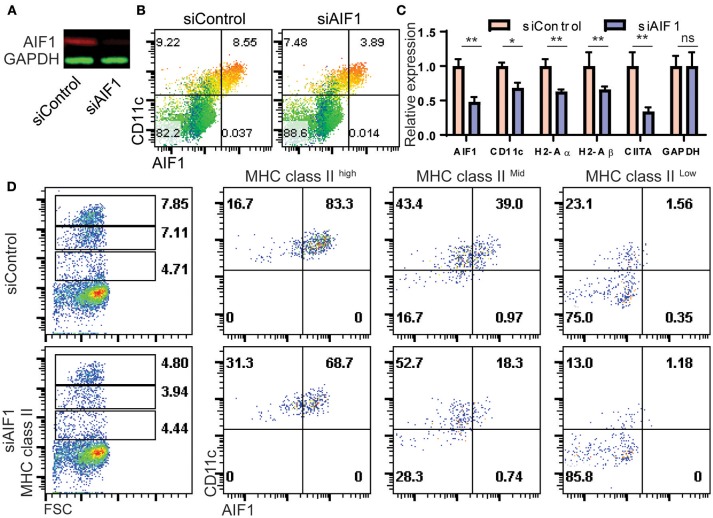
AIF1 silencing during GM-CSF-induced differentiation of bone marrow cells results in decreased MHC class II and CD11c expression. **(A)** Western blot analysis was performed on GM-CSF treated bone marrow cells silenced for AIF1 (siAIF1). Cells were collected and probed for AIF1. GAPDH served as an internal loading control. **(B)** Dot plots show flow cytometric analysis of AIF1 protein co-expression with CD11c using a heat map gradient relative to MHC class II. Heat-mapping is a visual technique which assigns a red-blue gradient to a range of data, varying the color based on the current cells' value relative to the distribution (with red as high and blue as low). **(C)** mRNA relative expression for AIF1, CD11c, H2-Aα, H2-Aβ, CIITA, and GAPDH was quantified in siControl and siAIF1 groups and plotted as a bar graph. **(D)** Flow cytometric analysis of AIF1 co-expression with CD11c in MHC class II ^high^, MHC class II ^middle^, or MHC class II ^low^ gated subsets. Data is representative of three independent experiments. Error bars for all figures indicate standard errors; ^*^< 0.05, ^**^< 0.01 and NS, not significant.

To determine if AIF1 was responsible for controlling CD11c^+^ Mo-DC development under GM-CSF stimuli, expression was silenced in bone marrow cells cultured over the 8-day differentiation period. Cells were transfected with siRNA targeting AIF1 (siAIF1) or control scrambled oligos (siControl) on day 0 of the differentiation process. Western blot analysis confirmed successful knockdown of AIF1 (Figure 4A). Upon assessing generation of Mo-DC on day 4, the earliest time point to which AIF1 expression was strongly observed under GM-CSF stimulation, results revealed reduction of CD11c^+^AIF1^+^ DC from 8.55% ± 0.4 in the control group to 3.89% ± 0.3 in the AIF1 knockdown group (Figure 4B). However, less impairment in generation of CD11c^+^AIF1^−^ subsets was observed, with a reduction from 9.22% ± 0.3 in the siControl compared to 7.48% ± 0.3 in the siAIF1 groups, respectively. Gene expression analyses revealed MHC class II (H2-Aα and H2-Aβ), CIITA and CD11c were all restrained upon silencing of AIF1 (Figure 4C). To corroborate transcriptional profile analyses, flow cytometry was performed. Results revealed that expression of both MHC class II^high^ and MHC class II^middle^ subsets were restrained in the AIF1 silenced cohort, but less effect was seen in the MHC class II^low^ population (Figure 4D). In particular, AIF1 was found to be most expressed in CD11c^+^MHC class II^high^ DC in comparison to the MHC class II^middle^ or MHC class II^low^ subsets. Taken together, these results suggest that generation of CD11c^+^MHC class II^+^ Mo-DC are dependent on AIF1 during *in vitro* GM-CSF differentiation from bone marrow precursors.

### Loss of AIF1 Restrains Commitment of Hematopoietic Stem Cell Progenitors Into cDC1 Subsets Through Repression of IRF8 and Batf3 Under Flt3-L Stimuli

**Figure 5:**
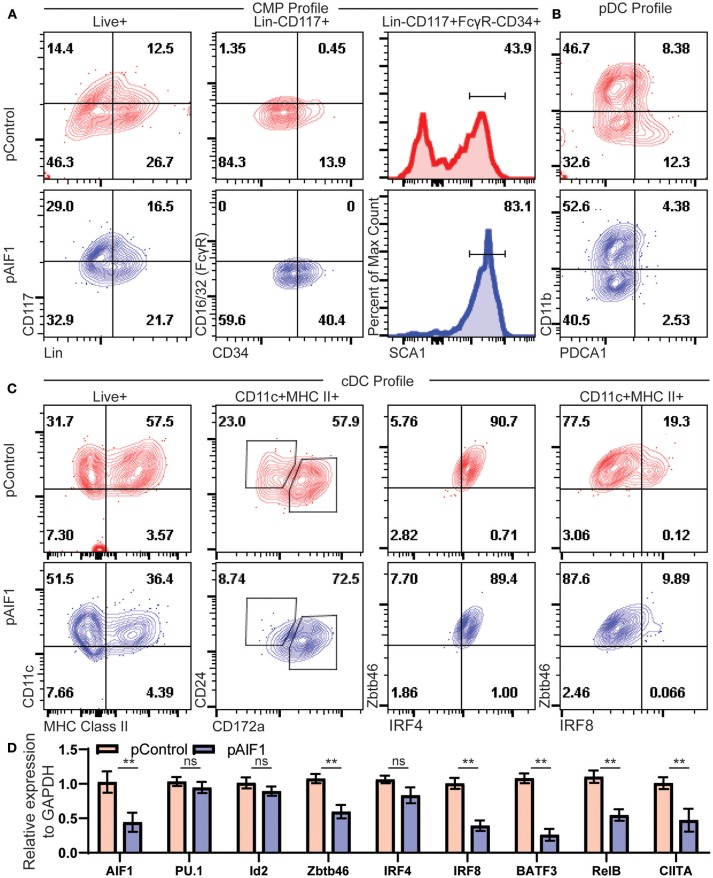
Absence of AIF1 restrains hematopoietic progenitor differentiation into cDC1 lineage commitment. Bone marrow cells were harvested and silenced for AIF1 by CRISPR RNAi approaches prior to differentiation into DC using Flt3-L as stimuli for 9 days. CRISPR Cas9 plasmids with either gRNA targeting AIF1 (pAIF1) or non-targeting scrambled gRNA (pControl) were used to transfect the cells. Cells were collected and stained with respective antibodies and Live/Dead viability dye to discriminate and gate on the Live^+^ population. **(A)** To assess common myeloid progenitor (CMP) profiles, dot plots show SCA1 expression in cells gated on Live^+^Lin^−^CD117^+^FcγR^−^CD34^+^ subset. **(B)** CD11b vs PDCA1 was utilized to assess pDC, where pDC are CD11b-PDCA1^+^. **(C)** cDC profile shows CD11c vs. MHC class II gated on total Live^+^ cells. CD24 vs CD172a were evaluated on the gated CD11c^+^MHC class II^+^ subsets. Next, cells were assessed for Zbtb46 vs. IRF4 or IRF8. **(D)** Real-time PCR analysis was performed on control or AIF1 silenced group to assess AIF1, PU.1, Id2, Zbtb46, IRF4, IRF8, BATF3, RelB, and CIITA. Data is representative of six independent experiments. Error bars for all figures indicate standard errors; ^**^< 0.01 and NS, not significant.

To more accurately assess cDC generation from hematopoietic precursors, expression of AIF1 was silenced in bone marrow cells by transfection with plasmids carrying the CRISPR-Cas9 with guide RNA targeting the gene at exon 7. Transfected cells were then cultured with Flt3-L for 9 days. Cells were then evaluated for expression of hematopoietic stem cell and progenitor markers by flow cytometric analyses. Results revealed a higher proportion of Lin^−^CD117^+^ in the AIF1 silenced group (pAIF1), having an increase from 14.4% ± 0.8 from the CRISPR-Cas9 control (pControl) to 29.0% ± 1.4 (Figure 5A). Next, the Lin^−^CD117^+^ cells were gated on to discretely assess levels of CD16/32 (FcγR) vs. CD34 to determine changes in proportion of CMP, megakaryocytic-erythroid progenitors, and granulocyte-monocyte progenitors (GMP). Results found no expression of FcγR associated with GMP subsets, as expected under Flt3-L treatments. However, there was a significant increase in the Lin^−^CD117^+^FcγR^−^CD34^+^ subsets from 13.9% ± 1.2 in the control group to 40.4 ± 0.8 upon AIF1 silencing (Figure 5A). Further analyses discovered increased SCA1 expression in the Lin^−^CD117^+^FcγR^−^CD34^+^ gated subset from 43.9% ± 1.4 in control to 83.1% ± 1.7 in pAIF1 transfected cohorts (Figure 5A).

Studies next aimed to investigate changes in classical vs. plasmacytoid DC (pDCs) generation. There was a significant decrease in CD11b^−^PDCA1^+^ subsets from 12.3% ± 2.6 to 2.53 ± 1.2, indicative of a reduction in the pDC compartment (Figure 5B). Most notably, loss of AIF1 restrained differentiation of CD11c^+^MHC class II^+^ DC from 57.5% ± 3.8 to 36.4% ± 4.1 upon AIF1 silencing (Figure 5C), recapturing observations in the Mo-DC studies. Further delineation revealed that AIF1 silencing under Flt3-L stimulation dominantly suppressed cDC1 development, but not cDC2. Evaluation of the CD11c^+^MHC class II^+^ subsets showed a decrease in the frequency of CD24^+^CD172α^−^ DC (cDC1) from 23.0% ± 0.8 to 8.74% ± 1.2, with an increase of the CD24^+^CD172α^+^ population from 57.9% ± 0.8 up to 72.5% ± 2.2. Next, CD11c^+^MHC class II^+^ DC were further analyzed for Zbtb46 co-expression, which serves as a marker to clearly identify classical DC, with IRF4 or IRF8. Results revealed no changes in IRF4 expression. However, there was a significant decrease in IRF8 within the CD11c^+^MHC class II^+^Zbtb46^+^ subsets from 19.3% ± 0.8 to 9.89% ± 0.5. Further delineation of key transcription factors associated with cDC and pDC differentiation were assessed by quantitative PCR (qPCR). Results showed down regulation of Zbtb46, IRF8, BATF3, RelB, and CIITA, but revealed no significant changes in PU.1, IRF4, or Id2 gene expression (Figure 5D). Collectively, these results show that restrained AIF1 expression in hematopoietic progenitors suppresses differentiation into distinct cDC1 subsets, while concomitantly redirecting toward cDC2 under Flt3-L stimuli.

### AIF1 Is Important for Committing Lin^−^CD117^+^ Hematopoietic Stem Cell Progenitors Into Mo-DC Subsets Under GM-CSF Stimuli

Bone marrow cells (pre-sort) were sorted for Lin^−^CD117^+^ progenitors (post-sort) to assess HSC ability to convert into Mo-DC (through the MDP-to-cMoP pathway) (Supplemental Figure 3A). Sorted Lin^−^CD117^+^ hematopoietic cells were then transfected with CRISPR-Cas9 targeting knockout of AIF1 prior to culturing with GM-CSF *in vitro* to generate Mo-DC. Silencing of AIF1 was successful using two different gRNAs targeting AIF1 (Supplemental Figure 3B). Results paralleled that of Flt3-L studies. The percentage of Lin^−^CD117^+^FcγR^−^CD34^+^ subsets was largely increased in absence of AIF1 during Mo-DC generation (Supplemental Figure 3C). This suggests that absence of AIF1 restrains conversion into DC subpopulations at the CMP/MDP stage during GM-CSF-stimuli conditions (Supplemental Figure 3D).

### Loss of AIF1 Impairs RelB Expression and Phosphorylation of p38 MAPK

**Figure 6:**
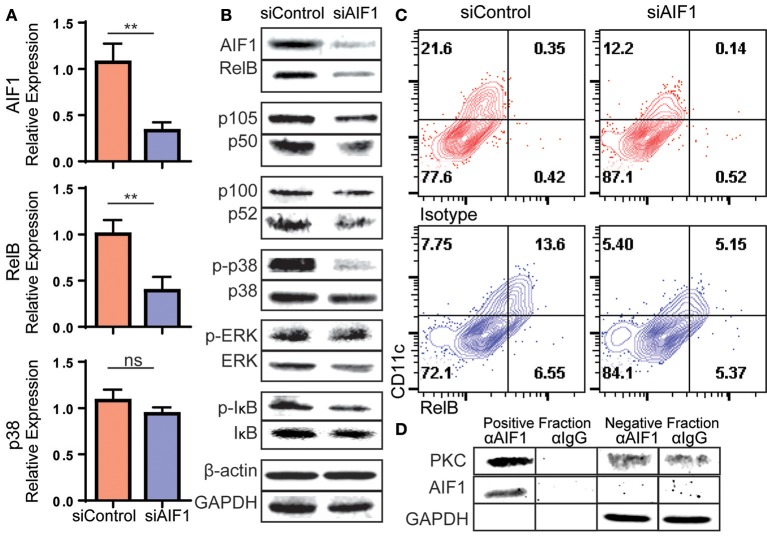
Loss of AIF1 inhibits RelB expression and p38 signaling during GM-CSF-induced DC differentiation. Murine bone marrow cells were sorted for Lin^−^CD117^+^ hematopoietic progenitors. These cells were silenced for AIF1 prior to stimulation with GM-CSF to generate DC. Cells harvested on day 6 were assessed by **(A)** qPCR for gene expression profiling of AIF1, RelB, and p38 or **(B)** western blot for protein expression of AIF1, RelB, p105/50, or p100/52. GAPDH and β-actin served as internal loading controls and for normalization. For evaluating phosphorylation of proteins, phosphorylated-IκB (p-IκB), -p38 (p-p38), and -ERK (p-ERK) were measured and normalized to total IκB, p38, and ERK protein, respectively. **(C)** Flow cytometric dot plots of control or AIF1 siRNA treated cells show RelB vs. CD11c. Gates were established using isotype controls. **(D)** Protein-protein interaction of AIF1 and PKC from DC were assessed. The precipitated positive fraction was pulled out with αAIF1 and further probed for PKC. IgG antibody served as isotype internal control. Negative fraction is the flow through. All data is representative of four independent experiments. Error bars for all figures indicate standard errors; ^**^< 0.01 and NS, not significant.

The NFκB and MAPK signaling pathways are important for DC development from hematopoietic stem cells. In particular, upregulation of the NFκB family member RelB is essential for differentiation of CD11c^+^ DC subsets. Loss of RelB results in deficient DC numbers and functioning *in vivo* (21, 22, 32). To investigate deregulated signaling events in absence of AIF1 during DC differentiation *in vitro*, changes in NFκB and MAPK family mRNA gene expression levels were measured. Silencing of AIF1 resulted in relative decreased expression of RelB (Figure 6A). Interestingly, there was no significant change observed in total p38 MAPK mRNA levels.

In measuring protein levels, results corroborated successful knockdown of AIF1 (down to 47.8% ± 0.9) from controls by western blot analysis. This was concomitant with a significant decrease in RelB down to 35.9% ± 0.9 in comparison to control DC (Figure 6B). This recaptured the observed fate seen in mRNA quantitative analysis. Interestingly, there were no changes in protein levels of the NFκB family member p105/50 (NFκB_1_) or p100/p52 (NFκB_2_) upon silencing of AIF1. Next, MAPK family proteins were evaluated. In particular, prior studies have shown that AIF1 is involved in governing p38 signaling activities (33). These studies corroborated those findings, whereby phosphorylation of p38 (phos-p38) was significantly reduced down to 57.3% ± 0.8 from controls upon AIF1 silencing (Figure 6B); levels were normalized to total p38. However, studies found no significant differences in that of phosphorylated-ERK signaling. Lastly, studies found reproducibly a moderate reduction in phosphorylated-IκB (phos-IκB); levels were normalized to total IκB.

Flow cytometric analysis was utilized to corroborate the observed decrease in RelB concomitantly with CD11c in absence of AIF1. Results revealed a decrease from 13.6% ± 0.7 of CD11c^+^RelB^+^ in control DC down to 5.1% ± 0.4 upon silencing of AIF1 on day 4 (Figure 6C). Given that PKC has been shown to promote RelB expression during DC differentiation (34) and AIF1 serves as a calcium responsive scaffold protein, co-immunoprecipitation was performed to detect potential protein-protein interactions. Results revealed that PKC was found to co-precipitate with AIF1 (Figure 6D), suggesting that AIF1 may serve as a docking site for recruiting and binding the protein kinase. Taken together, this AIF1-PKC interaction may be responsible for promoting NFκB (through phosphorylation of IκB) and MAPK signaling to drive RelB expression for successful differentiation of CD11c+MHC class II+ DC from hematopoietic progenitors.

## Discussion

The small proportion of AIF1 expressed in the bone marrow cells were largely restricted to the Lin^−^CD117^+^SCA1^+^CD34^−^ subsets. In contrast, the spleen expressed levels of the protein in both DC and macrophage populations. AIF1 was largely found in CD11c^+^MHC class II^+^ DC, with the majority expressed in CD8α^+^ cDC1 subsets. Notably, a significant amount of AIF1 was also expressed in the CD8α^−^ DC subsets. However, little-to-no expression was observed in lymphoid-derived subsets such as B and T cells or pDC, suggesting that AIF1 expression is restricted to the cDC and macrophage subsets.

Increased AIF1 levels were found in DC cultured *in vitro* from bone marrow cells under GM-CSF or Flt3-L stimuli conditions. Both approaches generated a population of CD11c^+^MHC class II^+^ subsets that expressed AIF1, where it was largely found in the monocyte-derived DC (expanded by GM-CSF) and cDC1 group (expanded by Flt3-L). This directly corroborates the observation of splenic cDC1 subsets dominantly expressing AIF1. Further analyses revealed that AIF1 is not present in all CD11c^+^MHC class II^+^ subsets, which supports why not all CD11c^+^ subsets (i.e., cDC2 expanded by the Flt3-L or the CD11b^+^CD11c^+^ monocyte-derived pool under GM-CSF stimuli) are restrained from development upon AIF1 silencing during differentiation.

Silencing AIF1 in bone marrow cells prior to *in vitro* differentiation by either GM-CSF or Flt3-L revealed its importance in directing DC commitment. Both siRNA and CRISPR-Cas9 approaches were used to successfully restrain AIF1 expression. To compensate for dilution of siRNA due to the accelerated proliferation rate of differentiating cells *in vitro*, and potentially weed out artifacts, CRISPR-Cas9 system was used to corroborate phenotypes. Both RNAi systems yielded an increase in the MDP and/or CDP in absence of AIF1 upon either GM-CSF or Flt3-L stimulation. These findings would suggest that in absence of AIF1, progenitors are partly being restrained from moving into subsets of monocyte-derived DC or cDC1 committed fates; thus, cells are being restrained at the earlier progenitor stage.

However, AIF1 seems to have a differential role in coordinating development of Mo-DC and cDC under GM-CSF- vs. Flt3-L-stimulated cultures, respectively. Under GM-CSF *in vitro* differentiation, absence of AIF1 was directly correlated with depressed levels of IRF4, BATF3, and CIITA. Less affect was reproducibly found on IRF8 and Zbtb46 expression. However, under Flt3-L conditions, there was largely reduced levels of Zbtb46, IRF8, BATF3, with no significant difference in IRF4 expression in cDC. This may be due to differential role and transcriptional machinery directing Mo-DC vs. cDC. For example, Mo-DC require IRF4 to induce MHC class II expression (35). Thus, AIF1 silencing could plausibly restrain IRF4 that leads to diminished MHC class II expression in the observed Mo-DC. However, the IRF8-BATF3 axis is required for coordinated cDC1, but not cDC2 fates (13, 36, 37), from Flt3-L stimulated cultures, which represent bona-fide *in vivo* DC counterparts. Interestingly, previous studies showed that loss of IRF8 lead to reduced expression levels of AIF1 in microglial cells (38). This further suggests bidirectional interactive roles of AIF1-IRF8 to govern myeloid differentiation and functions. However, AIF1 silencing did not perturb IRF4^+^ expression, which does align with no observed alteration in cDC2 fates. Therefore, AIF1 may influence/affect different transcriptional regulatory and downstream pathways that drive the respective fates of distinct DC subsets from Mo-DC vs. cDC. Coupled with literature eluding to the importance of monocytes and monocyte-derived DC as pivotal players of cross-priming events (39–41), this would suggest that AIF1 expression in myeloid cells is important for governing DC subsets involved in cross-presentation under steady and inflammatory settings.

For both cDC and Mo-DC, RelB expression was inhibited upon loss of AIF1. This would then restrict interactions with NFκB p52 complexes for downstream signaling. Given RelB is dispensable for *in vitro* Flt3-L cultures (23), but required for *in vivo*, studies focused on Mo-DC. In this respect, the proposed mechanism by which AIF1 governs DC differentiation may operate by additionally modulating RelB protein expression, as opposed to sequestering RelB activity through inhibitory protein-protein interactions with p100. Furthermore, results revealed an impaired ability to phosphorylate IκB in absence of AIF1. Reduced phosphorylation of IκB would inhibit activation and translocation of NFκB molecules for transcriptional control of RelB. Thus, it suggests that AIF1 silencing concomitantly restrains hematopoietic progenitor differentiation into Mo-DC or cDC1 subsets by disrupting NFκB transcriptional control of RelB (42).

Lastly, the studies identified that AIF1 interacts with PKC, which is an important upstream modulator of IKK phosphorylation of IκB and MAPK signaling (19). RelB activity is increased in absence of IκB-mediated activities (24). As AIF1 has been reported to function as an adaptor protein, which could explain its role in early signaling events. This correlates with prior studies that have shown that AIF1 associates with Rac2 and p38 MAPK (43), which are involved in maturation and cross-presentation (25, 44, 45). Therefore, we propose that AIF1 serves as a scaffold protein interacting with PKC to promote IRF8, BATF3, and RelB in cDC1 and RelB, CIITA, and IRF4 in Mo-DC during differentiation from HSC. In absence of AIF1, differentiation of these Mo-DC or cDC1 subsets from hematopoietic stem progenitors is abrogated.

## Author Contributions

DE, NB, RdS, and ML contributed in experimental design and performing experiments. NH, TdM, and NB performed qPCR assays. AK generated approaches for siRNA and CRISPR mediated transfections. DE, NB, RdS, NH, AK, TdM, and ML were each involved in data analysis and writing of the manuscript.

### Conflict of Interest Statement

The authors declare that the research was conducted in the absence of any commercial or financial relationships that could be construed as a potential conflict of interest.

## Abbreviations

AIF1: Allograft Inflammatory Factor-1
BM: bone marrow
cDC: classical dendritic cell
CDP: common dendritic progenitor
CMP: common myeloid progenitor
HSC: hematopoietic stem cell
LK-HSC: Lineage^−^ CD117^+^ hematopoietic stem cells
MDP: macrophage, monocyte dendritic progenitors
Mo-DC: Monocyte-derived dendritic cell
PKC: protein kinase C.
