# Supplementary figures and images for: Allograft Inflammatory Factor-1 Governs Hematopoietic Stem Cell Differentiation Into cDC1 and Monocyte-Derived Dendritic Cells Through IRF8 and RelB in vitro
Source: Front Immunol. 2019 Feb 8;10:173. doi: 10.3389/fimmu.2019.00173 (PMC6375893; doi:10.3389/fimmu.2019.00173)

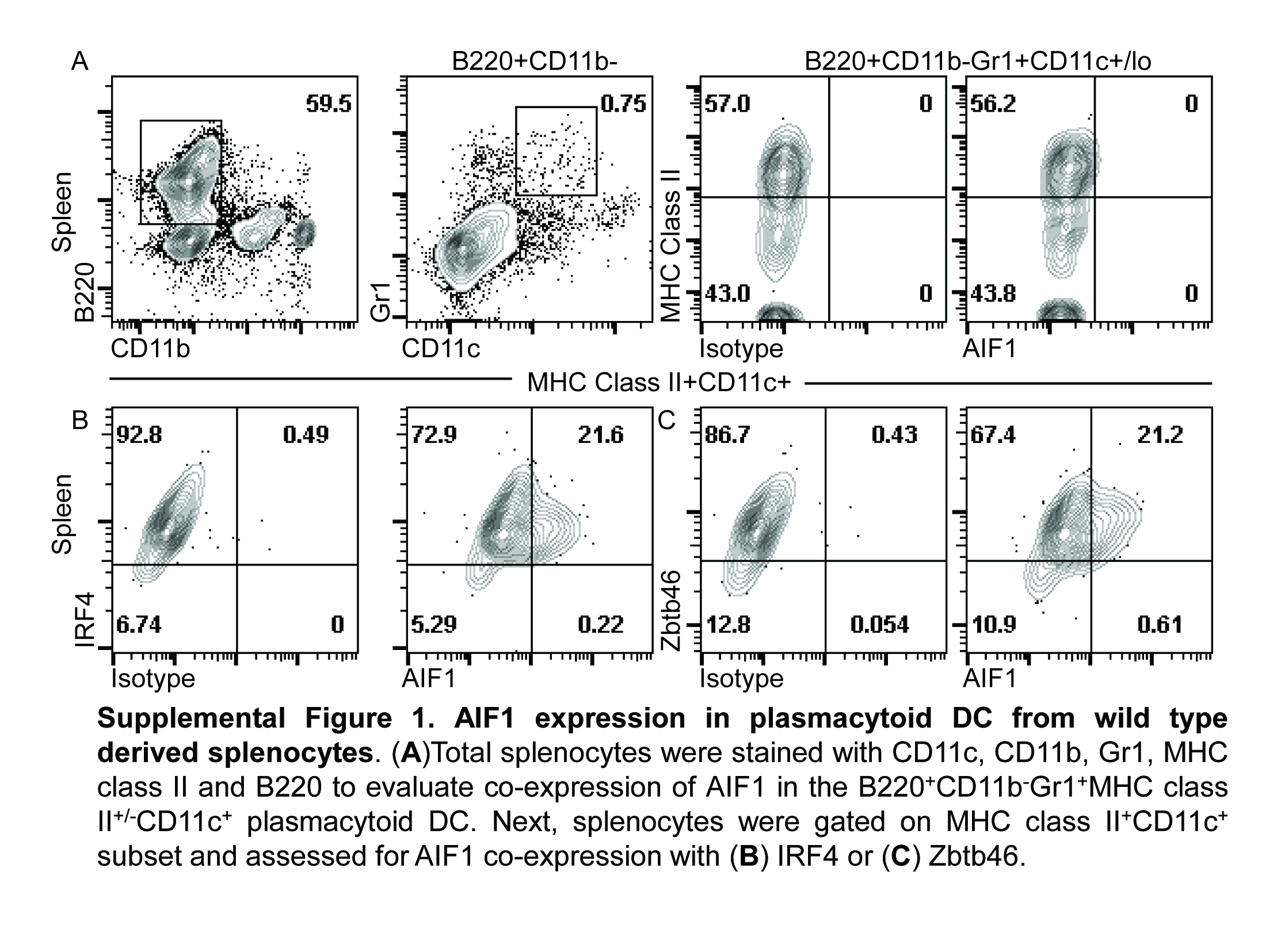

Supplement: Supplementary file 1 [file Image_1.TIF]

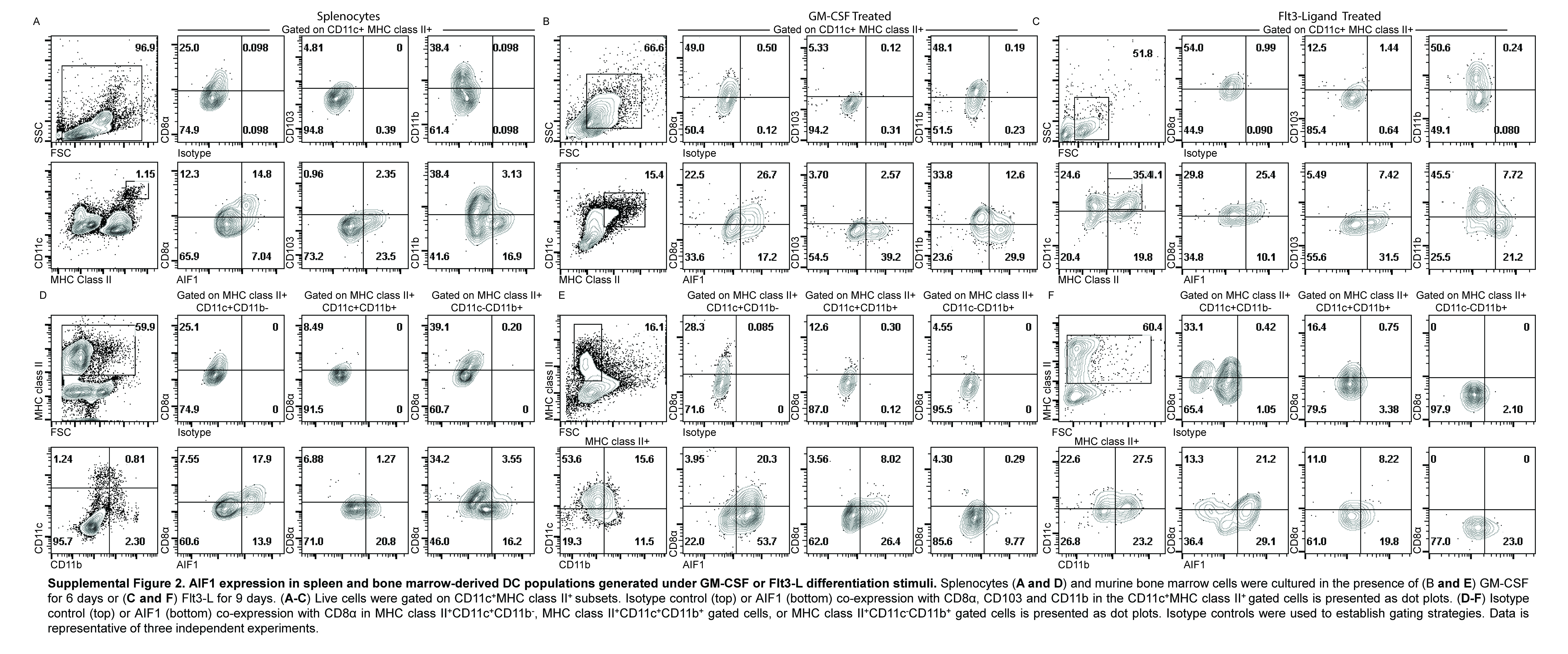

Supplement: Supplementary file 2 [file Image_2.TIF]

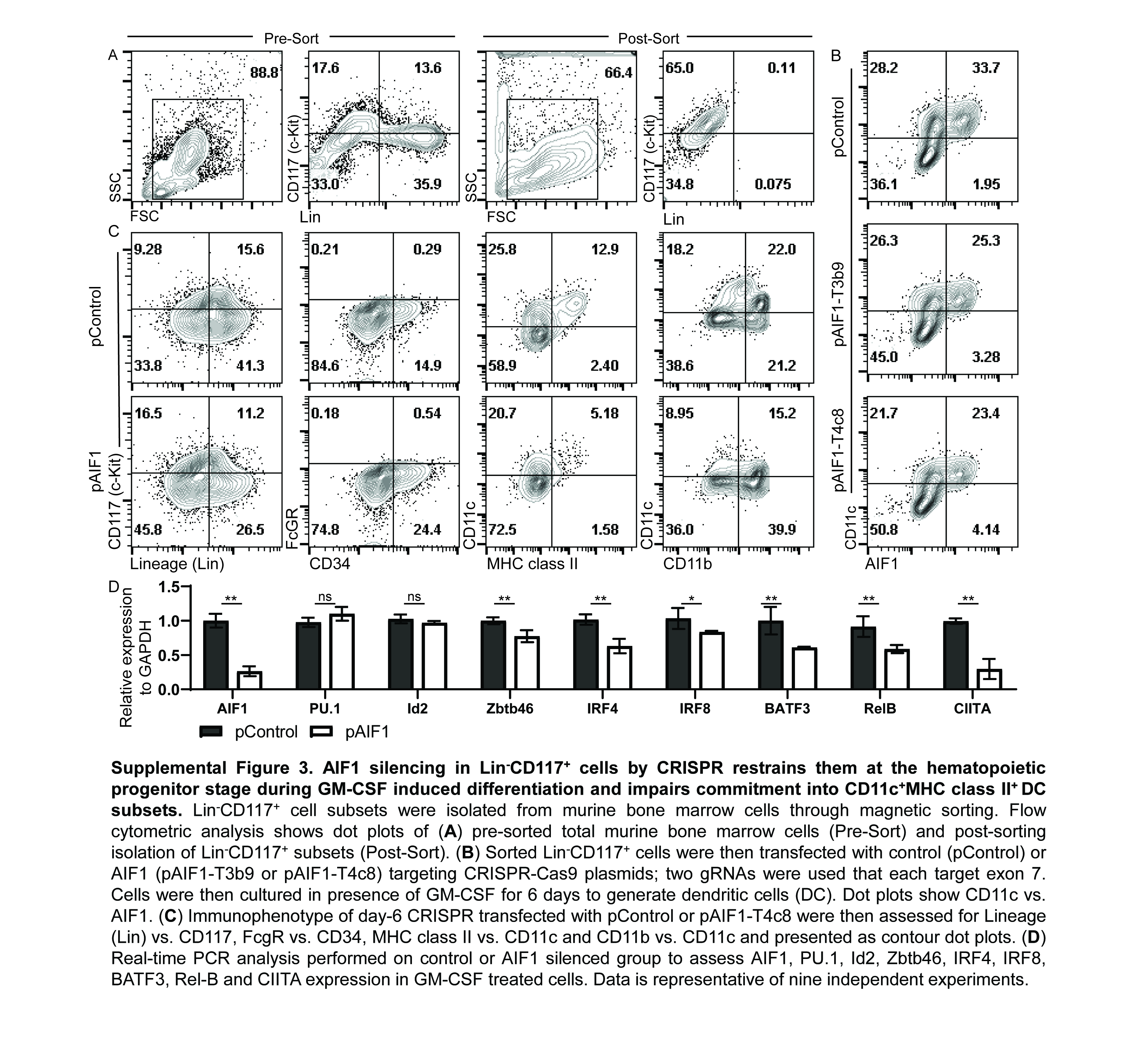

Supplement: Supplementary file 3 [file Image_3.TIF]
